# Valproic Acid Reduces Vasospasm through Modulation of Akt Phosphorylation and Attenuates Neuronal Apoptosis in Subarachnoid Hemorrhage Rats

**DOI:** 10.3390/ijms22115975

**Published:** 2021-06-01

**Authors:** Chieh-Hsin Wu, Yi-Cheng Tsai, Tai-Hsin Tsai, Keng-Liang Kuo, Yu-Feng Su, Chih-Hui Chang, Chih-Lung Lin

**Affiliations:** 1 Division of Neurosurgery, Department of Surgery, Kaohsiung Medical University Hospital, Kaohsiung 80756, Taiwan; wujoeys@gmail.com (C.-H.W.); Teishin8@hotmail.com (T.-H.T.); geet810224@yahoo.com.tw (K.-L.K.); suyufeng2000@gmail.com (Y.-F.S.); chchang20@gmail.com (C.-H.C.); 2 Department of Surgery, School of Medicine, College of Medicine, Kaohsiung Medical University, Kaohsiung 80756, Taiwan; 3 Graduate Institute of Medicine, College of Medicine, Kaohsiung Medical University, Kaohsiung 80756, Taiwan; iiidns11@hotmail.com

**Keywords:** apoptosis, Akt, endothelial nitric oxide synthase (eNOs), ERK, subarachnoid hemorrhage (SAH), valproic acid (VPA), vasospasm

## Abstract

Aneurysmal subarachnoid hemorrhage (SAH) is a devastating emergent event associated with high mortality and morbidity. Survivors usually experience functional neurological sequelae caused by vasospasm-related delayed ischemia. In this study, male Sprague-Dawley rats were randomly assigned to five groups: sham (non-SAH) group, SAH group, and three groups with SAH treated with different doses of valproic acid (VPA) (10, 20, 40 mg/kg, once-daily, for 7 days). The severity of vasospasm was determined by the ratio of cross-sectional areas to intima-media thickness of the basilar arteries (BA) on the seventh day after SAH. The BA showed decreased expression of phospho-Akt proteins. The dentate gyrus showed increased expression of cleaved caspase-3 and Bax proteins and decreased expression of Bcl-2, phospho-ERK 1/2, phospho-Akt and acetyl-histone H3 proteins. The incidence of SAH-induced vasospasm was significantly lower in the SAH group treated with VPA 40 mg/kg (*p* < 0.001). Moreover, all groups treated with VPA showed reversal of the above-mentioned protein expression in BA and the dentate gyrus. Treatment with VPA upregulated histone H3 acetylation and conferred anti-vasospastic and neuro-protective effects by enhancing Akt and/or ERK phosphorylation. This study demonstrated that VPA could alleviate delayed cerebral vasospasm induced neuro-apoptosis after SAH.

## 1. Introduction

Aneurysmal subarachnoid hemorrhage (SAH) occurs in a minority of strokes [1,2] but is a devastating neurologic event associated with mortality and morbidity rates exceeding 50% [3,4]. It affects relatively young patients, and one-third of survivors are rendered dependent due to major disability requiring costly medical care [2]. Early brain injury is the main cause of death in SAH [5] while vasospasm-related delayed ischemia causes 40% of deaths in patients who survive acute aneurysm rupture [6,7,8]. Outcomes in survivors are poor; more than 50% of survivors suffer long term cognitive or neurological deficits [9,10].

Ecker and Riemenschneider [11] first described the relationship between vasospasm and SAH in 1951, and cerebral vasospasm is now recognized as a proximate cause of delayed neurological deficit, ischemia and infarction [12,13]. Vasospasm develops 4 to 9 days after hemorrhage in 30% to 70% of SAH patients [14]. Both human and animal models have demonstrated apoptosis during aneurysm formation and rupture [15,16]. Granule cell apoptosis in the dentate gyrus has been detected in patients dying from SAH [17], and cell death observed in the vasculature and blood-brain barrier indicates that apoptotic cascades may be liable for vasospasm [18]. Zhou et al. further demonstrated in dog models of SAH that caspase inhibitors decrease endothelial apoptosis and vasospasm [19]. Cell death following SAH has important effects not merely for vasospasm, but also for sustained sequelae of SAH [18]. Although large trials have focused on vasospasm and its sequelae in recent decades, the rate of success in improving outcomes is low [20].

Valproic acid [2-propylpentanoic acid] (VPA), a class I/II histone deacetylase (HDAC) inhibitor of short-chain fatty acids [21], easily crosses the blood–brain barrier and is well recognized as the safest and most easily tolerated anti-convulsant and mood stabilizer [22]. Valproic acid is also commonly prescribed for treatment of neuropathic pain and migraines [23,24]. Additionally, VPA reportedly influences neurotransmission regulation and intracellular pathway accommodation during cell apoptosis, differentiation and growth [25]. In rat models, Zhang et al. showed that VPA may up-regulate activity of extracellular signal-regulated kinase (ERK) and protein kinase B (Akt) and may reduce the rate of neuronal apoptosis during the acute phase of traumatic brain injury [26]. Other preclinical studies have demonstrated neuro-protective properties in rat models of ischemic stroke [27,28] and neurodegenerative disease [29,30]. However, their association with VPA in cerebral vasospasm has not been fully illustrated.

Therefore, this study used a rat model of SAH to investigate whether VPA attenuates cerebral vasospasm or exerts a neuroprotective effect.

## 2. Results

### 2.1. Mortality

In total, 40 rats were used for in vivo studies. Each animal with SAH had a thick subarachnoid clot over the basal surface of the brain stem. On day 7 after the first SAH, all experimental rats had survived.

### 2.2. VPA Attenuated Vasospasm after SAH

**Figure 1:**
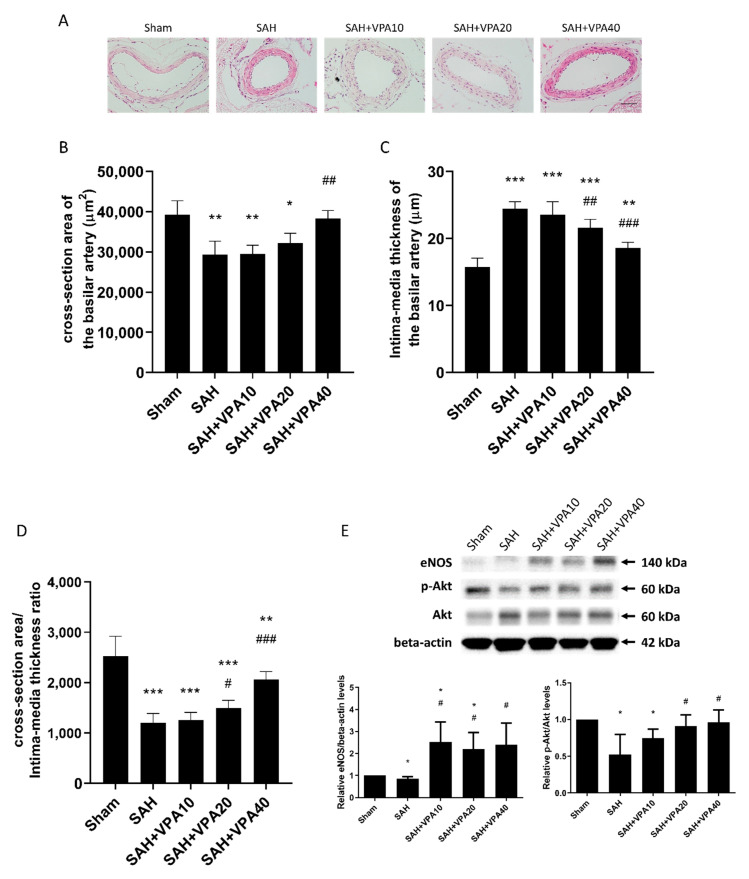
VPA ameliorated vasospasm in SAH rats. Comparison of cross-sectional areas of the lumen in basilar arteries (BA). (**A**) Representative cross-sectional micrographs of BA. (**B**) Quantification of cross-sectional areas of the lumen. (**C**) Quantification of intima-media thickness. (**D**) Quantification of the ratio of cross-sectional areas to the intima-media thickness. All values are expressed as mean +SEM (*n* = 5–7); * *p* < 0.05. ** *p* < 0.01, *** *p* < 0.001 in comparison with the sham group; # *p* < 0.05. ## *p* < 0.01, ### *p* < 0.001 in comparison with SAH group (scale bar = 50 μm) (**E**) Top panel: representative expression of eNOS, Akt and phospho-Akt protein of BA. Beta-actin was used as control. Lower panel: quantification of relative eNOS and *p*-Akt activity. All gels were run in the same experimental conditions (*n* = 3–4). * *p* < 0.05, compared with the sham group, # *p* < 0.05, compared with the SAH group.

To measure the effect of VPA in SAH rats, they were divided into five groups: sham (non-SAH) group, SAH group, and three groups with SAH treated with different doses of valproic acid (VPA) (10, 20, 40 mg/kg, once-daily, for 7 days). Sham-operated rats behaved as controls. Figure 1A shows that the cross-sectional areas of BA were significantly reduced in animals with SAH. The mean cross-sectional areas of the BA were 29,301.4 ± 3377.7 μm^2^ in the SAH group, 29,423.8 ± 2466.3 μm^2^ in the SAH-VPA10 group, and 31,666.8 ± 2407.0 μm^2^ in the SAH-VPA20 group. The mean cross-sectional area was 39,904.8 ± 3906.5 μm^2^ in the sham group. In comparison with the sham group, the mean cross-sectional areas of BA were significantly lower in the SAH group (26% lower; *p* < 0.01), in the SAH-VPA10 group (26% lower; *p* < 0.01), and in the SAH-VPA20 group (20% lower; *p* < 0.05). The mean cross-sectional area in the SAH-VPA40 group (38,445.0 ± 2235.3 μm^2^) significantly differed from that in the SAH group (*p* < 0.01). That is, the treatment with VPA (40 mg/kg, once-daily, for 7 days) achieved a statistically significant (*p* < 0.01) protective effect in comparison with the SAH group, and the mean cross-sectional area of the BA in the SAH-VPA40 group did not significantly differ from that in the sham group (Figure 1B). Moreover, the significant increase in the intima-media thickness after SAH was significantly attenuated by VPA (Figure 1C). Finally, the severity of vasospasm was determined by the ratio of cross-sectional areas to intima-media thickness of the BA. VPA (SAH-VPA40 group) significantly improved the ratio of cross-sectional areas to intima-media thickness, which was decreased after SAH (Figure 1D).

To clarify the aforementioned morphological change in the BA, Western blot analyses of BA were performed to examine Akt, phospho-Akt and endothelial nitric oxide synthase (eNOS). The expression of eNOS and phosphorylation of Akt proteins in BAs were significantly reduced after SAH. VPA treatment significantly reversed the reduced protein expression in the SAH groups, which suggests that VPA might prevent SAH-induced vasospasm through upregulating eNOS expression via the phosphorylation of the Akt signaling pathway (Figure 1E).

### 2.3. VPA Reversed SAH-Induced Mitochondrial Apoptosis Signaling in Dentate Gyrus

**Figure 2:**
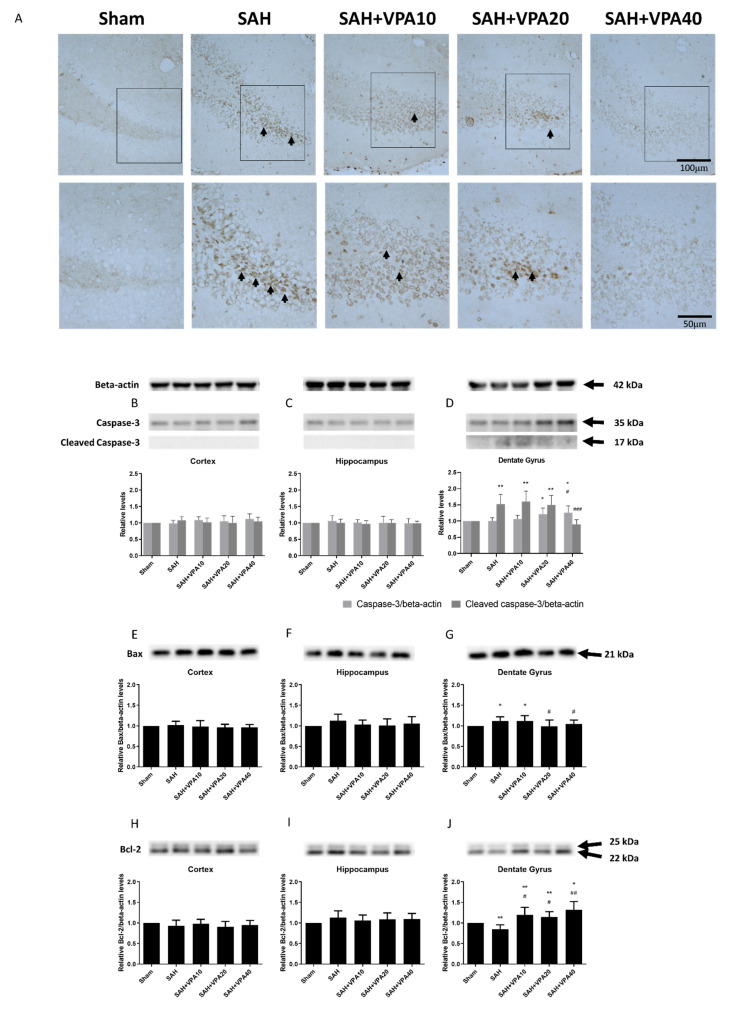
VPA reversed SAH-induced apoptosis signaling in dentate gyrus. (**A**) TUNEL labeling was used to identify apoptotic cells after SAH in response to VPA administration (scale bar = 100 μm). SAH induced neuronal apoptosis in the dentate gyrus whereas VPA decreased apoptotic cells (black arrows). Black rectangle in top panels indicates the apoptotic cells for high magnifications in lower panels. (scale bar = 50 μm) (**B**–**D**) Representative expression of caspase-3, cleaved caspase-3, (**E**–**G**) Bax and (**H**–**J**) Bcl-2. Beta-actin was used as control. All gels were run in the same experimental conditions (*n* = 6). * *p* < 0.05, ** *p* < 0.01, compared with the sham group, # *p* < 0.05, ## *p* < 0.01, ### *p* < 0.001, compared with the SAH group.

Fatal cases of SAH also exhibit apoptosis in the dentate gyrus [17]. In order to assess apoptotic cells in brain tissue, terminal deoxynucleotidyl transferase (TdT)-mediated dUTP nick end labeling (TUNEL) was employed. No apoptosis appeared in the sham group (Figure 2A). Seven days after SAH, apoptotic cells developed only in the dentate gyrus, not in the cortex and hippocampus. VPA treatment decreased the apoptosis in the SAH groups including SAH-VPA10, SAH-VPA20 and SAH-VPA40.

Next, the study examined protein expression of caspase-3 and cleaved caspase-3-in the cortex, hippocampus, and dentate gyrus 7 days after SAH. Western blot analysis revealed that SAH significantly initiated apoptosis in the mitochondrial pathway. Apoptotic cell death in the dentate gyrus was detected by Western blotting of cleaved caspase-3 expression. Cleaved caspase-3 levels in the dentate gyrus were significantly higher in the SAH group compared with the sham group (*p* < 0.01; Figure 2D), but those in the cortex (Figure 2B), hippocampus (Figure 2C) and sham with VPA group were not (Supplementary Materials, Figure S1A). However, protein levels of cleaved caspase-3 in the SAH-VPA40 group significantly (*p* < 0.001) decreased to the levels observed in the sham group.

Moreover, the Western blot results showed that expression of the Bax (pro-apoptotic) protein in the dentate gyrus significantly increased after SAH (*p* < 0.05; Figure 2G. Expression of the Bcl-2 (anti-apoptotic) protein in the dentate gyrus also significantly decreased after SAH (*p* < 0.01; Figure 2J). Compared to the SAH group, the SAH-VPA20 group and the SAH-VPA40 group had significant downregulation of Bax protein expression (*p* < 0.05; Figure 2G) whereas the SAH-VPA10, SAH-VPA20 and SAH-VPA40 groups all had significant upregulation of Bcl-2 expression (*p* < 0.05, *p* < 0.05, *p* < 0.01, respectively, Figure 2J). However, the sham group and the SAH group did not significantly differ in the expression of Bax and Bcl-2 in the cortex (Figure 2E,H) and hippocampus (Figure 2F,I). Compared to the sham group, the expression of Bax and Bcl-2 in sham with VPA group revealed no significant change (Supplementary Materials, Figure S1B,C)

### 2.4. VPA Enhanced ERK1/2 and Akt Activation in the Dentate Gyrus

**Figure 3:**
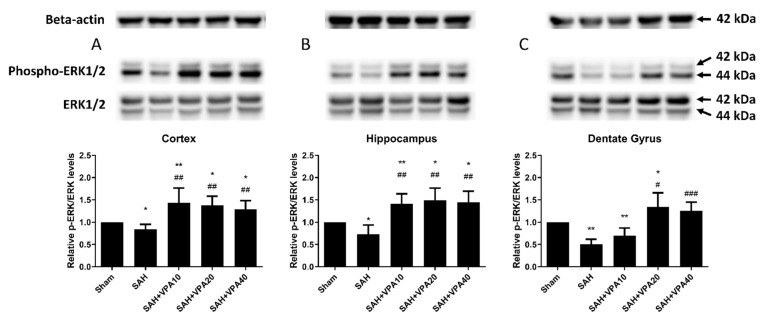
VPA enhanced ERK1/2 in brain tissue. (**A**–**C**) Top panel: representative expression of ERK 1/2 and phospho-ERK 1/2 proteins. Beta-actin was used as control. Lower panel: Quantification of relative *p*-ERK activity. All gels were run in the same experimental conditions (*n* = 6). * *p* < 0.05, ** *p* < 0.01, compared with the sham group, # *p* < 0.05, ## *p* < 0.01, ### *p* < 0.001, compared with the SAH group.

**Figure 4:**
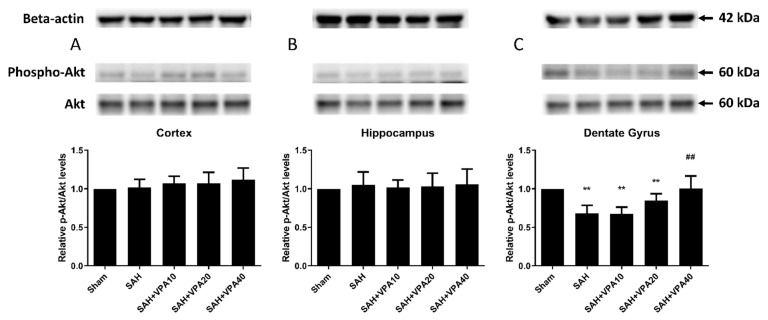
VPA upregulated Akt activation in dentate gyrus. (**A**–**C**) Top panel: representative expression of Akt and phospho-Akt protein. Beta-actin was used as control. Lower panel: quantification of relative *p*-Akt activity. All gels were run in the same experimental conditions (*n* = 6). ** *p* < 0.01, compared with the sham group, ## *p* < 0.01, compared with the SAH group.

After SAH, phospho-ERK 1/2 expression significantly decreased in the cortex (*p* < 0.05; Figure 3A), in the hippocampus (*p* < 0.05; Figure 3B) and in the dentate gyrus (*p* < 0.01; Figure 3C). The SAH-VPA20 and SAH-VPA40 groups showed the opposite effect, i.e., significantly increased phosphorylation of ERK 1/2 in the cortex (*p* < 0.01; Figure 3A), in the hippocampus (*p* < 0.01; Figure 3B), and in the dentate gyrus (*p* < 0.05 and *p* < 0.001, respectively, Figure 3C). However, phospho-Akt expression only showed a significant decrease in the dentate gyrus (*p* < 0.01; Figure 4C), not in the cortex (Figure 4A) and hippocampus (Figure 4B). The SAH-VPA40 group showed the opposite effect, i.e., significantly increased Akt phosphorylation in the dentate gyrus (*p* < 0.01; Figure 4C), not in the cortex (Figure 4A) and hippocampus (Figure 4B). Compared to the sham group, the expression of phosphorylation of ERK 1/2 and Akt in sham with VPA group revealed no significant change (Supplementary Materials, Figure S1D,E). These results suggest that the neuroprotective effect of VPA is likely associated with activation of the mitogen-activated protein kinase (MAPK)/ERK signaling pathway as well as activation of the lipid kinase phosphatidylinositol 3-kinase (PI3K)/Akt signaling pathway.

### 2.5. VPA Up-Regulated Histone H3 Acetylation in Brain Tissues after SAH

**Figure 5:**
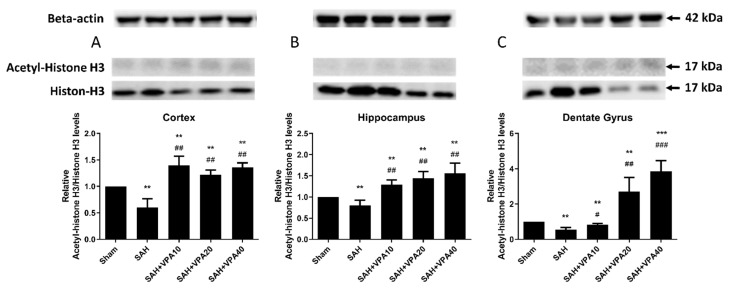
VPA attenuated SAH-induced hypoacetylation of histone H3 in brain tissue. (**A**–**C**) Top panel: representative expression levels of acetyl-histone H3 and histone H3 protein. Beta-actin was used as control. Lower panel: quantification of relative acetyl-H3 activity. All gels were run in the same experimental conditions (*n* = 6). ** *p* < 0.01,*** *p* < 0.001, compared with the sham group, ## *p* < 0.01,### *p* < 0.001, compared with the SAH group.

After SAH, acetyl-histone H3 expression significantly decreased in the cortex (*p* < 0.05; Figure 5A), hippocampus (*p* < 0.05; Figure 5B) and dentate gyrus (*p* < 0.01; Figure 5C). Treatment with VPA (10, 20, 40 mg/kg) significantly decreased hypoacetylation of histone H3 in the cortex (*p* < 0.01; Figure 5A), in the hippocampus (*p* < 0.01; Figure 5B), and in the dentate gyrus (*p* < 0.05; *p* < 0.01; *p* < 0.001; Figure 5C). Compared to the sham group, the expression of acetylation of histone H3 in the sham with VPA group revealed no significant change (Supplementary Materials, Figure S1F).

**Figure 6:**
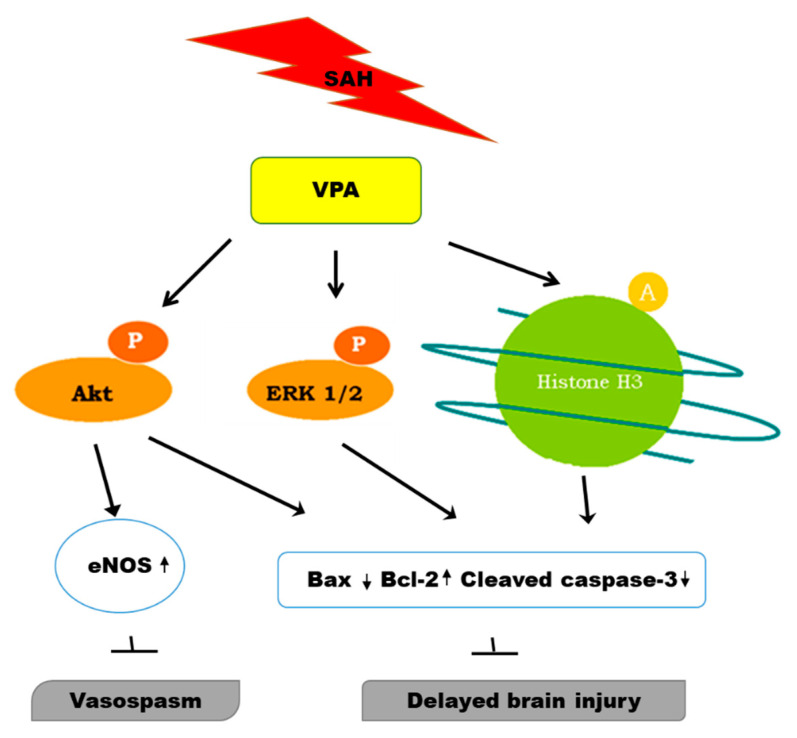
Hypothesized regulatory anti-vasospastic and neuroprotective role of VPA in SAH group.

These results suggest that VPA may alleviate SAH injury by acetylation of histone H3 and by phosphorylation of ERK/Akt, primarily in the dentate gyrus, and ameliorate vasospasm via phosphorylation Akt/eNOS signaling (Figure 6).

## 3. Discussion

In past decades, cerebral vasospasm has been considered the main cause of poor outcomes after aneurysmal SAH [20]. Cognitive dysfunction is a common disabling sequela [31]. In mature animals, neurogenesis develops in the dentate gyrus, a region of the brain important for the formation of new memories. In the absence of other hippocampal abnormalities, neuronal apoptosis in the dentate gyrus can cause impairment of memory and spatial learning [32]. Accumulating evidence suggests that cell death has crucial effects on vasospasm and is a key mediator of secondary brain injury after SAH and long-term sequela [18]. The present study found that VPA effectively attenuates SAH-induced cerebral vasospasm by upregulating eNOS expression and brain damage by decreasing apoptosis as measured by caspase-3 assays, in the dentate gyrus after SAH.

Different models of SAH reveal different time courses of vasoconstriction. In one study, for example, rat models of single or double SAH revealed biphasic vasospasms in the BA as early as 10 min after injection (early vasospasm) and as late as 2–7 days after blood injection (delayed vasospasm) [33]. In 2003, our research team reported low mortality in a mice model of double SAH [34]. In 2019, a rat model of double SAH with low mortality was used in our study as in previous research [35,36,37]. In the present study, we administrated VPA and euthanized the animals on day 7 after SAH when delayed phases of vasoconstriction occurred; the results indicated that VPA can prevent delayed cerebral vasospasm through upregulation of eNOS in BA.

Depending on the model and species used in the SAH model, SAH-induced apoptosis may be cell-specific and may have different effects [38,39]. Prunell et al. hypothesized that the death of cells adjacent to a blood clot resulted from direct toxic effects of the subarachnoid blood whereas global ischemia contributed to the death of cells remote from the blood clot after SAH [40]. In humans, apoptosis of neurons occurred specifically in the dentate gyrus when death occurred more than 24 h and less than 11 days after hemorrhage. In the rat model of double SAH in the present study, no deaths occurred, and thick clots accumulated over the basal surface of the brain stem. The characteristics of apoptosis in the dentate gyrus observed in the rat model were consistent with the actual area and timing of apoptosis in humans, probably because cisternal injection was performed.

A SAH can cause apoptosis in cortical, subcortical, and hippocampal neurons, in endothelia, and in the blood–brain barrier [19,41,42]. Mammals have three important pathways of mitochondria-related apoptosis [43], of which the intrinsic mitochondrial-dependent pathway is apparently the main contributor to apoptosis [44]. Of the 14 caspases that have been identified so far, caspase-3 is the crucial effecter in neuronal apoptosis [45]. Activated caspase 3 is strongly associated with apoptosis [46]. The pro-survival protein Bcl-2 regulates cytochrome c release by changing the permeability of the mitochondrial membrane. Ultimately, protein Bcl-2 decreases cleavage and activation of caspases and blocks Bax and Bad [18,47]. The ratio of Bcl-2 to Bax indicates whether cell survival or cell death is predominant. In the present study, increased expression of cleaved caspase-3 and Bax observed in the dentate gyrus after SAH were consistent with apoptosis. The VPA revealed an inhibiting effect on caspase-3 activation through the mitochondrial apoptotic pathway and blocked pro-apoptotic protein.

Earlier, Endo et al. reported that the distribution of Akt phosphorylation correlated with the severity of SAH. Additionally, Tibb et al. reported that MAPK inhibitors reversed cerebral vasospasm in a dog model of SAH [48,49]. Besides, since eNOS is important to generate nitric oxide, a vasodilator in SAH, it is activated through signaling of PI3K/Akt pathway in endothelial cells, in which phosphorylation of Akt is pivotal for eNOS activity [50,51]. Activation of the PI3K/Akt and the MAPK/ERK signaling pathway both contribute to neuronal survival [52,53,54]. The PI3K recruits Akt into the cellular membrane and then induces Akt phosphorylation at the Ser473 residue, which in turn phosphorylates and blocks Bad and NF-κB [51]. The MAPK signaling system comprises ERK, JNK, and p38 MAPK, which up-regulate Nrf2 signaling [55]. Activation of JNK and p38 MAPK and inactivation of ERK signaling are closely associated with cell apoptosis [56]. Moreover, activation of Akt or ERK can inhibit downstream glycogen synthase kinase-3b (GSK3b), which increases neuronal resistance to apoptosis [53,57]. Studies indicate that the ERK/Akt pathway contributes to the anti-apoptotic effect of neurotrophic factors [58,59]. To investigate possible mechanisms of VPA in vasospasm or brain damage, expression levels of eNOS, total and phosphorylated ERK and Akt (two pro-survival molecules) were investigated by Western blot analysis. In the present study, eNOS in BA, phosphorylation of Akt in BA and the dentate gyrus, and phosphorylation of ERK 1/2 in brain tissue decreased after SAH but were reversible by VPA. These observations may further suggest the possible roles of Akt in SAH-induced vasospasm, and Akt and ERK 1/2 in SAH-induced apoptosis.

The VPA confers neuro-protective effects through multiple mechanisms. Previous studies have shown that VPA is known to increase GABA-ergic transmission in rats and to alleviate spreading depolarizations, which is considered a cause of post-SAH delayed brain injury [60,61,62]. Since VPA is a histone deacelylase inhibitor [63], it directly regulates gene expression through hyperacetylation of promoter factors. Moreover, it can also selectively modulate transcriptional factors through hyperacetylation of non-histone proteins [64]. The acetylated histone H3 level in peripheral blood mononuclear cells reportedly has a negative association with the severity of cerebral infarction [65]. Acetylated histone protein confers neuro-protective effects by inhibiting neuronal cell death, which then improves neurological function in cerebral ischemia and traumatic brain injury [66,67]. Apart from inhibiting HDAC, VPA activates multiple signal transduction pathways, including PI3K/Akt, MAPK/ERK, GSK-3/β-catenin and Sp1/HSP 70 [24,27]. These signaling pathways affect cell growth, differentiation and apoptosis [21,68,69,70]. In Machado-Joseph disease, VPA reportedly enhances cell viability and neuroprotection by promoting H3 acetylation and activating transcription [71]. Thus, to identify changes in acetylation of histone H3 (an index of HDAC inhibition) after SAH, this study examined the ratio of acetyl-histone H3 expression to histone H3 expression in brain tissues. The present study found that by inhibiting histone deacetylase, VPA upregulates acetylation of histone H3 expression in brain tissue, but most obviously in the dentate gyrus. This suggests that histone deacetylation might play a role in suppressing neuronal apoptosis in SAH, which is parallel to the attenuation of vasospasm.

Although VPA is widely used to prevent epilepsy and bipolar disorder, the effects of VPA on SAH are less discussed in the literature. A study by the Loch Macdonald group [62] reported beneficial effects of VPA treatment in mice after SAH. Their randomized study indicated that VPA administration only improved neurobehavioral outcomes by decreasing degeneration of neurons in SAH mice and did not affect vasospasm. Different dose–response effects depend on different species of experimental animals. Larger animals required smaller drug dose on a weight basis as demonstrated in previous studies [72]. Therefore, our results showed that the effective dose (40 mg/day) for treating vasospasm in SAH rats is lower than those in the mice study (400 mg/kg). Furthermore, the prechiasmatic blood injection model of SAH used in that study differed from our animal model of SAH. Another difference is that the authors investigated the effect of every 12-h administration of VPA for 48 h after SAH. Additionally, they investigated possible mechanisms at 2 days after VPA administration whereas our study investigated possible mechanisms at 7 days after VPA administration. Another previous study [37] showed that vasospasm could be attenuated by expression of ICAM-1, E-selectin and RANTES. Expression of RANTES could promote trans-endothelial migration of monocytes through a chemokine ligand-5 (CCL5) dependent mechanism. However, the authors mention that continuous administration of VPA exerts anti-vasospastic effects by suppressing SAH-induced adhesion molecules and chronic inflammation. Male Sprague-Dawley (SD) rats weighing between 300–400 g received SAH in their study whereas we utilized male SD rats weighing 350–400 g. Besides, possible CCL5 dependent mechanisms at 5 days after VPA administration were clarified in their study whereas we elucidated delayed cerebral vasospasm-related mechanisms at 7 days after daily VPA administration. Previous studies have indicated that morphological changes in organs were based on different age and weight [73,74]. Therefore, depending on different animals (different weights) and sacrificial time, the degree of decreased BA cross-sectional areas varied, although BA areas were significantly reduced compared with the sham group in both studies. In another study by Hamming et al. [60], VPA treatment significantly reduced the growth of brain lesions after application of KCL to induce spreading depolarization through its anti-excitotoxic properties or by influencing hemodynamics. In their experiments, the authors observed that VPA had no protective effect when spreading depolarization was not induced. In the rat model of SAH used in that study, however, SAH was induced by intracranial endovascular perforation. Additionally, the authors focused on the effect of pretreatment with daily administration of VPA starting 4 weeks before induction of SAH. Thus, the aforementioned studies differed in their experimental protocol for administration of VPA (dosage/time or duration), in their animal models and in the time of cerebral vasospasm or injury.

First, vasospasm peaked earlier in the rat model than it would in humans [75,76]. In the rat model, double SAH caused a vasospasm mimicking the time course and pathological changes observed in human vasospasms. However, further studies are needed to determine whether these findings can be extrapolated to humans. Further animal studies are also needed to extend these observations. Second, brain tissues were examined at only one time-point (7 days after SAH) in this study; the long-term effects of VPA on survival and other SAH outcomes remain unknown. Third, although serum or cerebral spinal fluid (CSF) VPA levels were measured to correlate the therapeutic effect in epilepsy, the relevance of precise levels of rat serum or CSF VPA to anti-vasospastic effects were unknown in SAH. Although the current results show that higher-dose VPA improved the vasospasm at 7 days in SAH rats, the effects of different serum or CSF VPA levels on SAH outcome need further study.

## 4. Materials and Methods

### 4.1. Ethics Statement

All procedures using experimental animals were carried out in accordance with relevant ethical regulations and guidelines for the care and use of laboratory animals. The protocols were approved by the Institutional Animal Care and Use Committee of Kaohsiung Medical University. All efforts were made to minimize the suffering of animals. The study was carried out in compliance with the ARRIVE guidelines.

### 4.2. Animal Preparation

Male Sprague-Dawley male rats (body weight 350–400 g) were randomly divided into five groups (eight rats/group): (1) a sham group (non-SAH group); (2) a group with SAH (SAH group); (3) a group with SAH treated with VPA 10 mg/kg, once-daily for 7 days (SAH-VPA10 group); (4) a group with SAH treated with VPA 20 mg/kg, once-daily for 7 days (SAH-VPA20 group); and (5) a group with SAH treated with VPA 40 mg/kg, once-daily for 7 days (SAH-VPA40 group). The three groups treated with VPA received a single intraperitoneal injection of VPA 30 min after induction of SAH. Sham-operated rats behaved as controls. The animals were kept in a 12-h light/dark cycle with free access to food and water.

### 4.3. Experimental SAH Model

A rat model of double SAH was prepared as described previously [36,75,77]. Rats were anesthetized by intraperitoneal injection of Zoletil 50 (50 mg/kg, Virbac, Carros cedex). A rectal temperature of 36 ± 1 °C was maintained with a heating pad (Harvard Apparatus, Holliston, MA.). The head was fixed in a stereotactic frame in a nose-down position, and the parietal bone was tilted forward approximately 300 degrees in the superior plane. A percutaneous puncture was made in the cisterna magna with a 25-gauge butterfly needle [78]. Cerebrospinal fluid (0.1 to 0.15 mL) was slowly withdrawn, and the junction of the butterfly needle and tube was clamped. Autologous non-heparinized blood (0.3 mL) was withdrawn from the tail artery. Blood was slowly injected into the cisterna magna for 2 min by the needle-in-needle method performed with a 30-gauge needle inserted into a 25-gauge butterfly needle at the junction of the needle and tube. Five minutes after the injection, the butterfly needle was removed slowly to prevent leakage of injected blood. The rat was then removed from the stereotactic frame and positioned in the ventral recumbent position for 30 min to allow ventral blood clot formation [75]. The animal was then restrained, and the body temperature was maintained at 36 + 1 °C until full recovery. The same procedure was repeated 48 h later. The sham group underwent the same procedure with saline injected instead of blood. At 7 days after the first SAH, brain tissue samples were taken for analysis after normal saline infusion.

### 4.4. Sample Preparation

For basilar artery morphometric studies and TUNEL staining, the animals were sacrificed by perfusion with phosphate-buffered saline (PBS) and fixed with 4% paraformaldehyde. The rat brains were removed, postfixed in 4% paraformaldehyde for 24 h, and placed in 30% sucrose for 48 h. Then, the brain tissues were embedded in the Tissue-Tek^®^ optimal-cutting-temperature compound (Sakura Finetek Japan Co. Ltd., Koto-ku, Tokyo, Japan) and sliced into 10 μm-thick sections using a Cryostat Microtome (CM1800, Leica, Buffalo Grove, IL, USA).

For Western blot analysis, the animals were sacrificed by perfusion with PBS. The rat brains were removed and washed in ice-cool PBS. Basilar artery tissues were separated from the brain stem (from bregma-9 to bregma-15), and the whole brain tissues were cut into 2 mm coronal sections with a Rat Acrylic Brain Matrices (51384, Stoelting Co., Wood Dale, IL, USA). With the aid of a dissecting microscope (Nikon, Melville, NY, USA), the whole brain was separated into the cerebral cortex, the hippocampus and the dentate gyrus.

### 4.5. Basilar Artery Morphometric Studies

Morphometric measurements were utilized to measure the BA, of which the middle third part was dissected for analysis (continuous coronal frozen section 10 μm from bregma-10 to bregma-14). In each animal, at least six random arterial cross-sections were qualitatively evaluated for the extent of internal lumen. The arterial cross-sections and intima-media thickness were measured with a computer-assisted image analysis system [35,36,79,80,81,82]. For each animal, the area of the BA was defined as the average area for six cross-sections of the BA. The intima-media thickness was measured at the 3, 6, 9, and 12 o’clock positions of each segment of the BA. The ratio of cross-section area to the intima-media thickness was measured to assess the degree of vasospasm. Group data were expressed as mean ± standard error of the mean (SEM).

### 4.6. TUNEL Staining

Brain tissue was cut into 10 μm coronal sections. To determine of apoptotic cells, brain sections were stained with TUNEL staining using an in situ cell death detection kit, POD (Roche, Mannheim, Germany).

### 4.7. Western Blot Analysis

Western blot analysis was performed as described previously [50,73,75]. The experimental protocol included six animals in each group. Samples were obtained from the BA, cerebral cortex, hippocampus and dentate gyrus [74]. Tissue was homogenized in ice-cold T-PER Tissue Protein Extraction Reagent (Pierce Biotechnology, Inc., Rockford, IL, USA) with protease inhibitor (Complete Mini; Roche, Mannheim, Germany) and phosphatase inhibitor (PhosSTO; Roche, Mannheim, Germany) then centrifuged at 15,000 rpm for 20 min. Protein concentrations were estimated by Bio-Rad protein microassay. Samples were heated by immersion in boiling water for 5 min. Equal amounts of protein were loaded into each band of SDS-PAGE [50]. The gels were transferred onto polyvinylidene difluoride membranes by electroblotting for 90 min, and the membrane was blocked overnight at 4 °C with Tween-Tris buffer saline solution containing 5% nonfat dry milk and 0.1% Tween 20. The blot was incubated with primary antibodies eNOS (1:1000;BD; 610329), ERK1/2 (1:1000; Cell Signaling; 9102), phospho-ERK1/2 (1:500; Cell Signaling; 9211), Akt (1:1000; Cell Signaling; 9272), phospho-Akt (1:500; Cell Signaling; 9271), acetyl-histone H3 (1:500; Sigma; H0913), Histone H3 (1:1000; Sigma; 9715), Bax (1:2000; Proteintech; 50599-2-1g), Bcl-2 (1:500; BioLegend; 611901), caspase-3 (1:1000; Cell Signaling; 9662) and cleaved caspase-3 (1:400; Cell Signaling; 9661) and a 1:8000 dilution of the antibody against β-actin (Sigma; A5441). After a 30 min rinse with t-TBS, the membranes were incubated with secondary antibody conjugated to horseradish peroxidase (Jackson ImmunoResearch, West Grove, PA, USA). The membranes were then rinsed with t-TBS for 30 min, incubated with electrochemiluminescence reagent [75] (PerkinElmer, Waltham, MA, USA) for 2 min, and were visualized with the MiNiChemi Image System (Sage Creation Science Co., Ltd. Beijing, China). The intensity of each band was quantified by ImageJ software (National Institutes of Health, Bethesda, MD, USA). Beta-actin was used as control. Relative protein levels were normalized to beta-actin and calculated as the ratio of target protein to beta-actin. All data were normalized with the values of the sham group.

### 4.8. Statistical Analysis

The data were expressed as mean ± SEM. Two-way analysis of variance and the Bonferroni post-hoc test were used for group comparisons. A *p* value less than 0.05 was considered statistically significant.

## 5. Conclusions

This study showed that VPA treatment ameliorates cerebral vasospasm and alleviates delayed apoptosis in the dentate gyrus after SAH. Apoptosis induced by SAH occurs through a mitochondrial-dependent pathway. Treatment with VPA can activate the ERK and Akt pathways and can activate histone H3 acetylation. Our findings provide convincing evidence that VPA has both anti-vasospastic and neuro-protective effect, and hence, potential therapeutic applications for reducing delayed brain cells death after SAH.

## Data Availability

All data generated or analyzed during this study are included in this published article and its supplementary information files.

## Supplementary Materials

The following are available online at https://www.mdpi.com/article/10.3390/ijms22115975/s1.
