# Supplementary figures and images for: Valproic Acid Reduces Vasospasm through Modulation of Akt Phosphorylation and Attenuates Neuronal Apoptosis in Subarachnoid Hemorrhage Rats
Source: Int J Mol Sci. 2021 Jun 1;22(11):5975. doi: 10.3390/ijms22115975 (PMC8198375; doi:10.3390/ijms22115975)

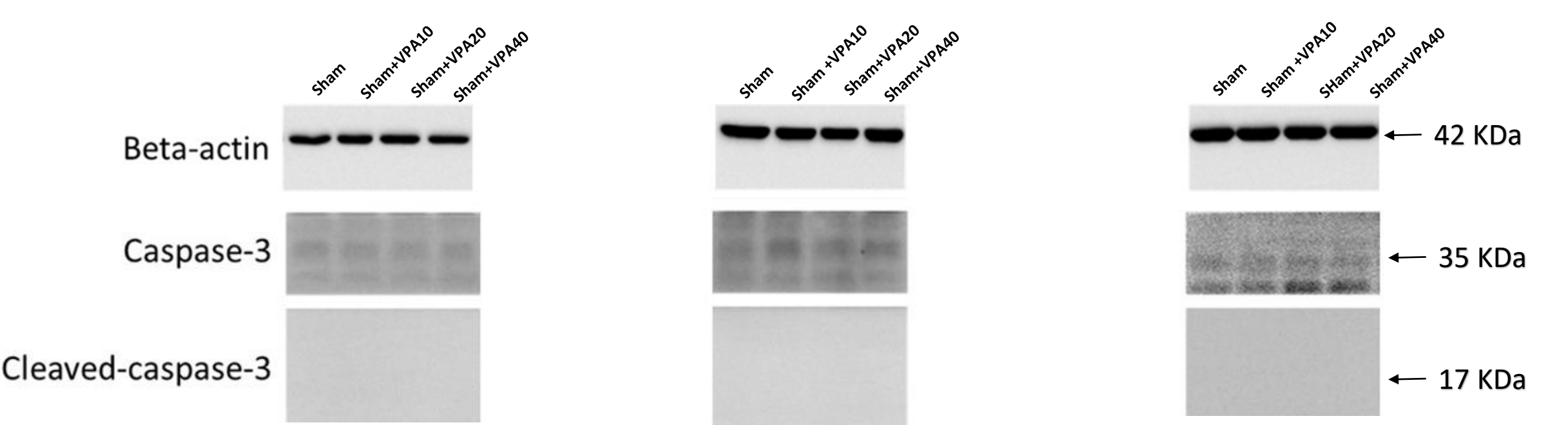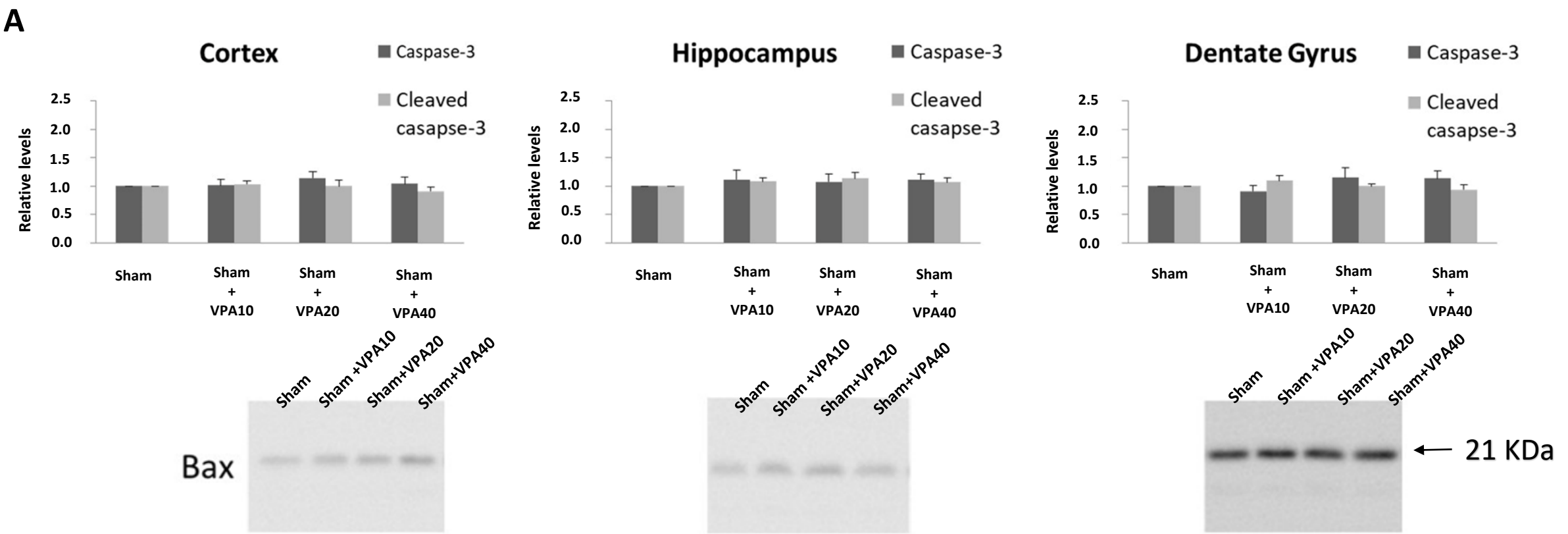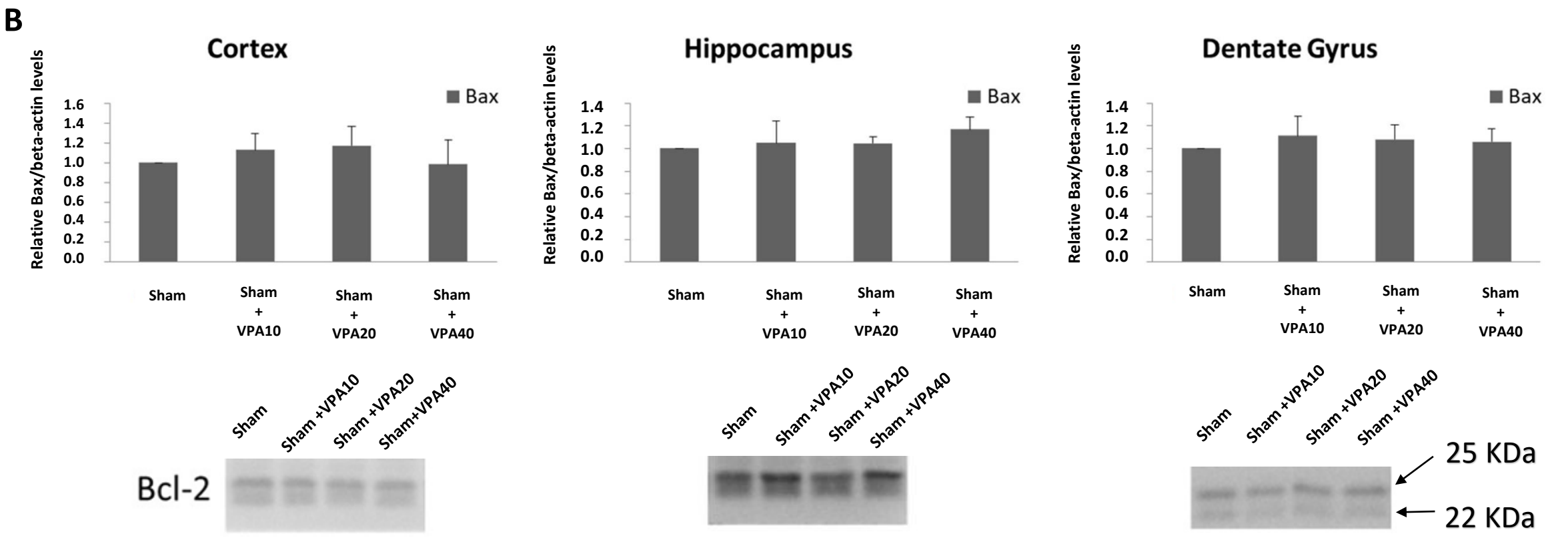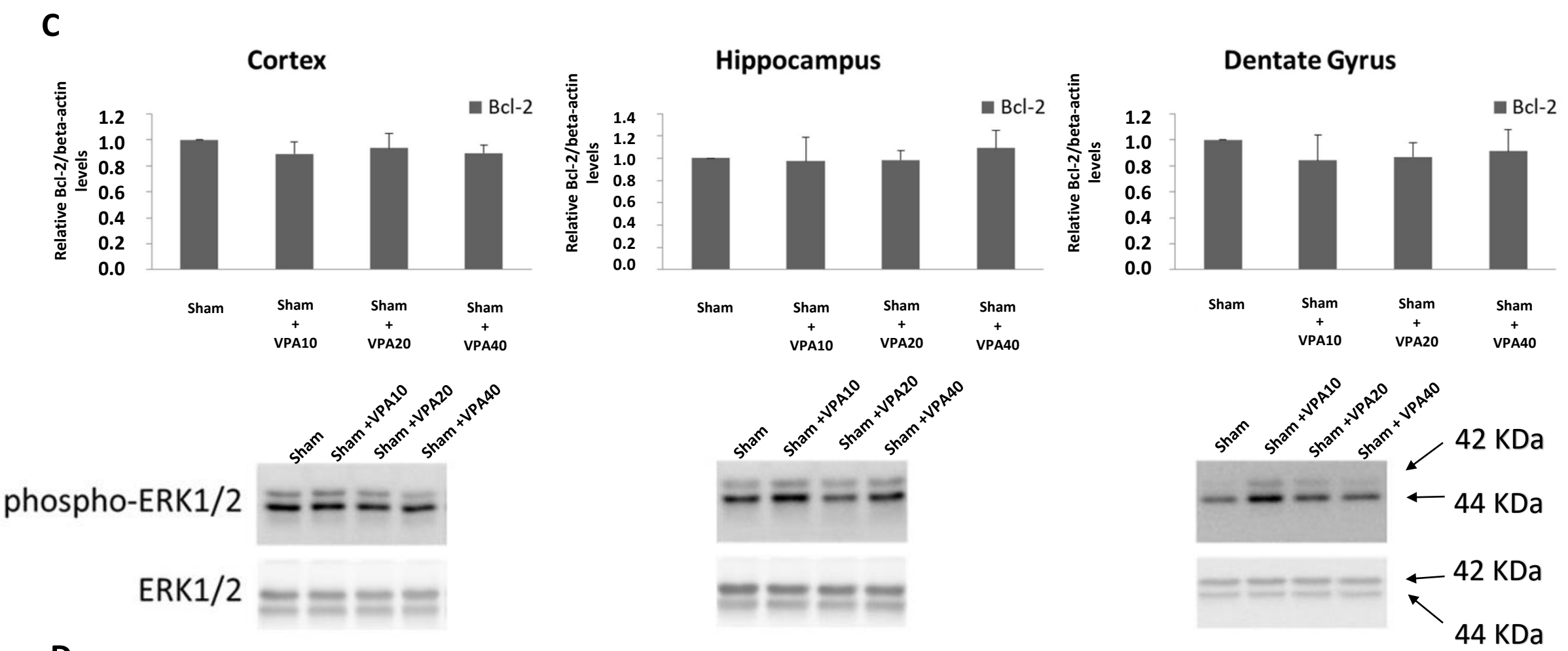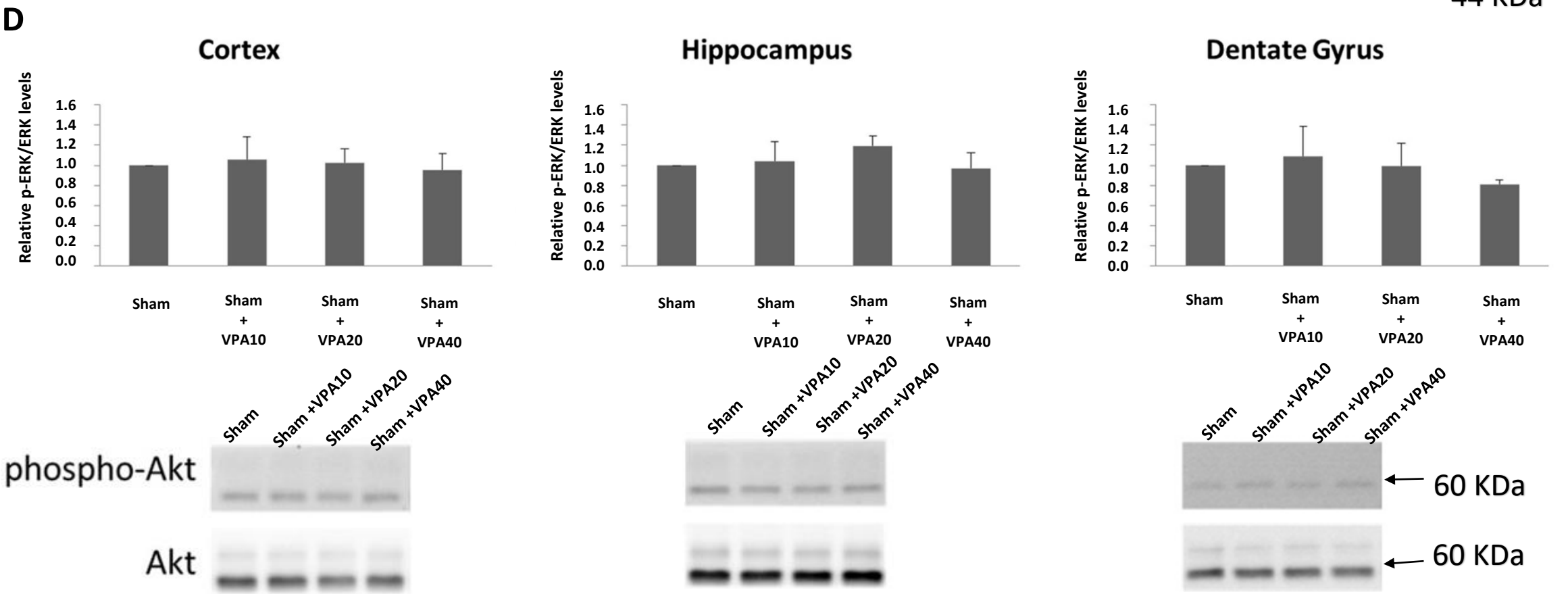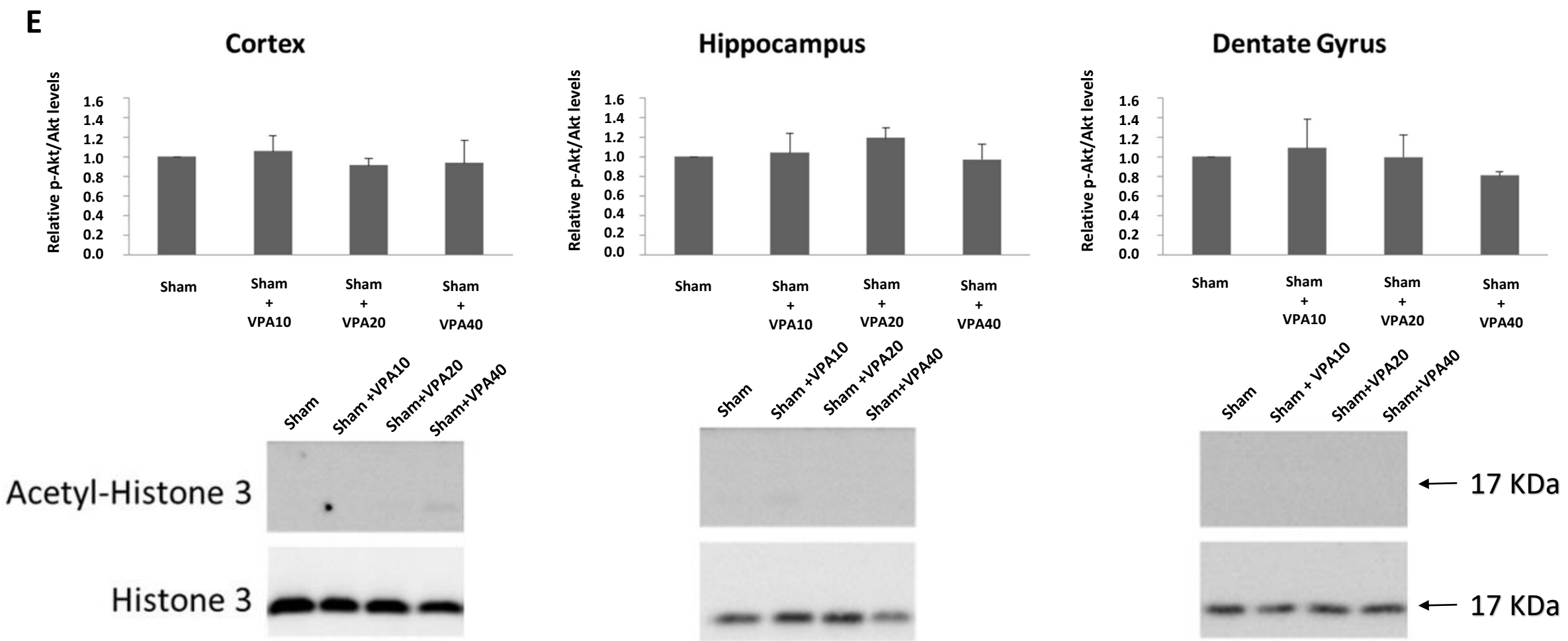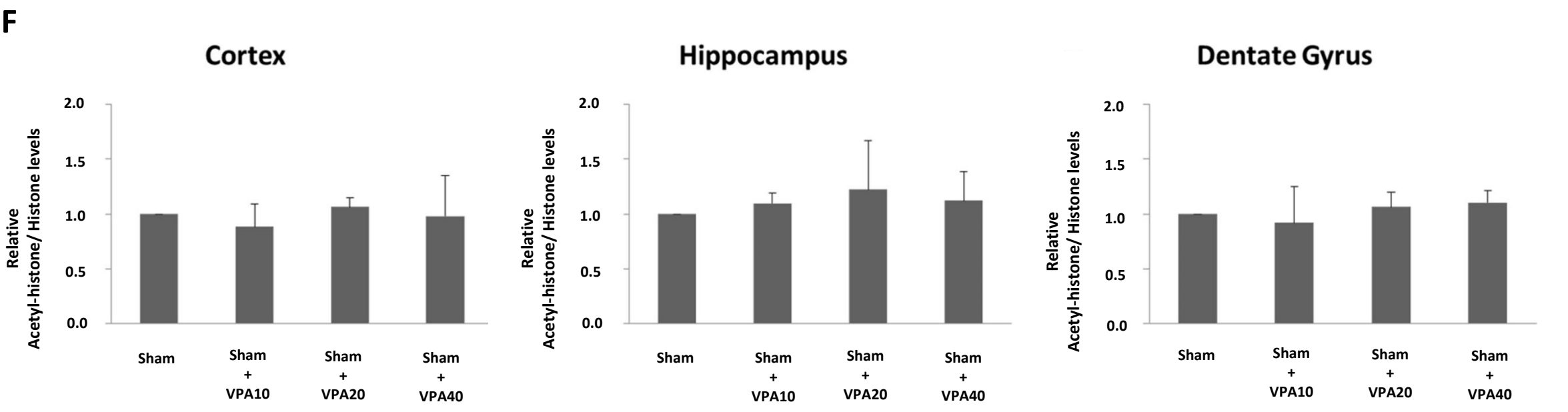

Supplement: Supplementary file 1 [file ijms-22-05975-s001.zip › ijms-1141938-supplementary.pdf]
